# Attitude toward second opinions in Germany – a survey of the general population

**DOI:** 10.1186/s12913-021-07422-z

**Published:** 2022-01-15

**Authors:** Nadja Könsgen, Barbara Prediger, Anna Schlimbach, Ana-Mihaela Bora, Simone Hess, Michael Caspers, Dawid Pieper

**Affiliations:** 1 Institute for Research in Operative Medicine, Witten/Herdecke University, Witten, Germany; 2 Faculty of Health Sciences Brandenburg, Brandenburg Medical School Theodor Fontane, Institute for Health Services and Health System Research, Neuruppin, Germany; 3 Center for Health Services Research, Brandenburg Medical School Theodor Fontane, Neuruppin, Germany

**Keywords:** Second opinion, Informed decision making, Patient autonomy, Patient preferences, Survey, General population

## Abstract

**Background:**

Second medical opinions (SOs) can assist patients in making informed treatment decisions and improve the understanding of their diagnosis. In Germany, there are different approaches to obtain a structured SO procedure: SO programs by health insurers and SOs according to the SO Directive. Through a direct survey of the population, we aimed to assess how structured SOs should be provided to fulfil patients’ needs.

**Methods:**

A stratified sample of 9990 adults (≥18 years) living in the federal states of Berlin and Brandenburg (Germany) were initially contacted by post in April and sent a reminder in May 2020. The survey results were analyzed descriptively.

**Results:**

Among 1349 participants (response rate 14%), 56% were female and the median age was 58 years (interquartile range (IQR) 44–69). Participants wanted to be informed directly and personally about the possibility of obtaining an SO (89%; 1201/1349). They preferred to be informed by their physician (93%; 1249/1349). A majority of participants would consider it important to obtain an SO for oncological indications (78%; 1049/1349). Only a subset of the participants would seek an SO via their health insurer or via an online portal (43%; 577/1349 and 16%; 221/1349). A personally delivered SO was the preferred route of SO delivery, as 97% (1305/1349) would (tend to) consider this way of obtaining an SO. Participants were asked to imagine having moderate knee pain for years, resulting in a treatment recommendation for knee joint replacement. They were requested to rate potential qualification criteria for a physician providing the SO. The criteria rated to be most important were experience with the recommended diagnosis/treatment (criterion (very) important for 93%; 1257/1349) and knowledge of the current state of research (criterion (very) important for 86%; 1158/1349). Participants were willing to travel 60 min (median; IQR 60–120) and wait 4 weeks (median; IQR 2–4) for their SO in the hypothetical case of knee pain.

**Conclusion:**

In general, SOs were viewed positively. We found that participants have clear preferences regarding SOs. We propose that these preferences should be taken into account in the future design and development of SO programs.

**Supplementary Information:**

The online version contains supplementary material available at 10.1186/s12913-021-07422-z.

## Introduction

Second medical opinions (SOs) can assist patients in making treatment decisions and increase the understanding of their diagnosis [1, 2]. In addition, they reduce the number of surgeries [3–7]. It has been found that they can lead to changes in diagnosis or treatment, even though there is some variation with regard to the rate of disagreements between first opinions and SOs [8, 9]. SO programs were originally introduced in the USA in the 1970s [4]. Currently, SOs are implemented in many countries [3, 10–13]. A survey of the German population found that 24% had already obtained one or more SOs [10].

In Germany, the right to obtain an independent SO free of charge was initially implemented by the SO Directive in 2019. This applies to individuals with statutory health insurance, making up a vast majority in the German population. The first medical indications included in the SO Directive were hysterectomy and tonsillectomy or tonsillotomy. The list of indications included in the SO Directive was later extended to shoulder arthroscopy, amputation of the lower extremities, knee endoprosthesis, and spine surgery. The list of indications will probably be extended further [14]. The SO Directives sets the framework within which SOs must be offered to individuals with statutory health insurance. The SO Directive is set up by a committee that includes representatives of service providers (physicians, dentists, hospitals) as well as statutory health insurers. According to the SO Directive, SOs for the included indications need to be provided verbally, thus allowing for telemedical SOs. Statutory health insurers may also provide SO programs for indications that are not part of the SO Directive. Many statutory health insurers provide (telemedical) SO programs [15]. These SO programs are often provided based on documents only and often exclude direct and personal contact between patients and physicians [16]. Even though high patient satisfaction was found for customers of an online portal providing SOs based on documents only [17], a survey of the general population in Germany found that 90% preferred personally delivered SOs [10]. This indicates that there are discrepancies between the provision of SOs by health insurers and the patients’ needs.

We aimed to survey the general population in Germany on their views of SO programs, and to identify diverging perspectives for people living in areas with diverse degrees of urbanization. We asked participants to compare SOs based on documents only with personally delivered SOs, and to name potential situations in which they would seek SOs based on documents only. This was done to characterize patient collectives and situations in which either type of SO provision would be appropriate, and to assess advantages and disadvantages. We further aimed to explore how SOs should be provided to best fulfil the patients’ needs. This included, but was not restricted to, qualification criteria for the physician providing the SO and acceptable waiting and travel times to obtain an SO.

## Methods

The survey was conducted as part of the ‘ZWEIT’ project funded by the ‘Innovation Fund’ of the Federal Joint Committee. More details on ZWEIT can be found in the study protocol [18]. Our survey was approved by the ethics committee of Witten/Herdecke University (case number: 162/2019). We followed the guidelines on conducting and reporting survey research [19]. The questionnaire was developed in multistage meetings between researchers. Its structure is shown in Table 1. Parts of the questionnaire were adapted from our previous survey of customers of a telemedical SO provider. This relates mainly to questions on sociodemographic characteristics and a few questions on patients’ experience with SOs [20]. Health literacy was assessed using the 16-item European Health Literacy Survey [21]. The answer category ‘do not know’ was added to the 4 answer options (very easy, fairly easy, fairly difficult, and very difficult). Furthermore, our questionnaire contained 4 items adapted (at least partially) from a previous survey on SOs [10]. Our questionnaire consisted of a mixture of open-ended and close-ended questions. For some of the close-ended questions, we provided dichotomous answers, whereas for others, we provided a list of possible answers. Some questions allowed for multiple answers, and others required a single answer. We piloted the questionnaire with a sample of 18 persons (heterogeneous in terms of sociodemographic characteristics).

**Table 1:** Structure of the questionnaire

Part	Number of items	Content
1	2	health-related items
2	5	local health care situation
3	7	patients’ needs concerning SOs
4	8	patients’ experiences with SOs
5	8	design of the SO procedure
6	3	experiences with and knowledge of SO programs by health insurers
7	3	experiences with and knowledge of the offer of SO providers
8	11	sociodemographic characteristics

In the first step, all local registration offices in Berlin and Brandenburg were listed and sorted by settlement pattern (cities, towns and suburbs, rural areas). For each settlement pattern, 10 local registration offices were randomly selected. Because there are only five local registration offices for the settlement pattern cities in Berlin and Brandenburg, we selected all of them. We asked the local registration offices to provide postal addresses of randomly selected adult residents (*n* = 333 each for the 10 local registration offices of towns and suburbs as well as of rural areas and *n* = 666 for local registration offices of cities). This process led to a 1:1:1 disproportionate stratified sampling based on settlement patterns (cities, towns and suburbs, rural areas). This sampling method was chosen to detect potential differences in the attitude toward SOs based on the settlement pattern. Finally, through the 25 local registration offices, we identified a stratified sample of 9990 adults (≥18 years) living in the federal states of Berlin and Brandenburg, Germany.

We initially contacted eligible persons by post in April 2020 and sent a reminder in May 2020. Because responders were asked to return the completed questionnaire, consent for participation was implicitly provided. Postage was paid by Witten/Herdecke University, and participants could win one of 125 Amazon vouchers amounting to 50€. The declaration of consent required to participate in the lottery was returned in a separate envelope. The questionnaire and consent form were separated immediately upon arrival. Therefore, no assignment to individuals was possible. One researcher extracted data from each questionnaire received into an Excel spreadsheet. Another researcher checked a random sample of 10% for accuracy. For open-ended questions, categorization by one author was verified by another author. We resolved discrepancies by discussion and, if necessary, by consulting another author. To analyze the extracted data, we used Microsoft Excel. We compared settlement patterns between all persons invited for participation and the participants. We categorized postal codes according to the ‘degree of urbanisation’ of Eurostat [22] and regional statistics by the Federal Statistical Office [23]. The Comparative Analysis of Social Mobility in Industrial Nations (CASMIN) categorization [24] was used to classify the participants’ education level. CASMIN contains three main categories (primary, secondary and tertiary education) and several subcategories. Primary education (inadequately completed general education) was not applicable to our sample. Therefore, we only considered the categories tertiary education (academic degree, independent of school education and type of degree), higher secondary education (no or any vocational education with at least intermediate school education) and lower secondary education (no or any vocational education with at least general school education).

We planned to perform a subgroup analysis comparing participants living in rural areas and participants living in cities. For continuous data, we planned to use a t-test to test for significant differences. In case of dichotomous data, we planned to use a chi-squared test.

## Results

### Basic characteristics

A total of *N* = 9797 were contacted successfully, and 193 invitations to participate were undeliverable within 2 attempts. Of the 9797 persons contacted, 1349 persons participated in our survey (14% response rate). Due to the study design, we were not able to systematically collect the reasons for nonparticipation.

When compared to all persons invited for participation, slightly more people who live in cities and slightly less who live in towns and suburbs participated. The basic characteristics of the participants are shown in Table 2.

**Table 2:** Basic characteristics

	%; n/N or median; interquartile range
Age	58 (44–69) years
Gender	
Female	56%; 758/1349
Male	43%; 580/1349
Diverse	0%; 0/1349
No (valid) answer	1%; 11/1349
Health insurance status	
Statutory	76%; 1023/1349
Private	7%; 98/1349
No (valid) answer	17%; 228/1349
Settlement pattern	
Cities	38%; 511/1349
Towns and suburbs	26%; 353/1349
Rural areas	33%; 443/1349
No (valid) answer	3%; 42/1349
Education level according to CASMIN	
Lower secondary education	10%; 130/1349
Higher secondary education	50%; 677/1349
Tertiary education	35%; 473/1349
No (valid) answer	5%; 69/1349
Number of household members	
Living alone	24%; 329/1349
Two or more household members	73%; 983/1349
No (valid) answer	3%; 37/1349
Health literacy	
(Likely) inadequate (0–8)	20%; 271/1349
(Likely) problematic (9–12)	33%; 447/1349
(Likely) sufficient (13–16)	30%; 399/1349
No (valid) answer	17%; 232/1349

### Patients’ needs concerning SOs

**Fig. 1:**
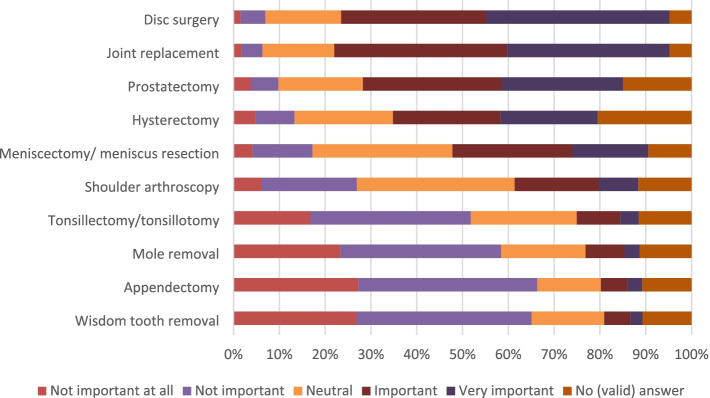
Rating of importance of SOs for specific procedures

While 33% (446/1349) never considered obtaining an SO in the past, 25% (341/1349) had contemplated it once and 38% (519/1349) twice or more. Participants generally found it important to obtain an SO for cancer diagnoses (78%; 1049/1349), diseases of bones, joints, and muscles (58%; 784/1349), diseases of internal organs (56%; 758/1349), neurological diseases (46%; 621/1349), and diseases of the mind/psyche (164/1349). The top 3 rated specific indications were joint replacement, disc surgery, and prostatectomy. In these indications, 73% (987/1349), 72% (967/1349), and 57% (767/1349) participants, respectively, stated that obtaining an SO would be (very) important (see Fig. 1). The preferred way to obtain an SO was via a physician in a practice (81%; 1090/1349) or a physician in a hospital (72%; 973/1349). Obtaining an SO via the health insurer or an online portal was less favored (43%; 577/1349 and 16%; 221/1349). By far the most popular method of SO delivery was direct and personal patient-physician contact (97%; 1305/1349 would (tend to) consider this way of SO delivery). Only few participants would (tend to) consider obtaining SOs via phone or based on documents only (23%; 314/1349, and 16%; 219/1349, respectively). Most participants rated SOs from direct and personal patient-physician contact better than SOs based on documents only (73%; 991/1349), 10% rated them equally (133/1349), and 1% rated them worse (19/1349). More detailed information can be found in Table 3 and in the Supplementary material.

**Table 3:** Main results

	%; n/N
Have you considered seeking a second opinion in the past? (multiple answers NOT possible)	
Yes, twice or more	38%; 519/1349
No, never	33%; 446/1349
Yes, once	25%; 341/1349
I do not know	3%; 35/1349
No (valid) answer	1%; 8/1349
Where can you generally imagine seeking a second opinion? (multiple answers possible)	
Physician in a practice	81%; 1090/1349
Physician in a hospital	72%; 973/1349
Via the health insurer	43%; 577/1349
Via an online portal	16%; 221/1349
Other	4%; 52/1349
No (valid) answer	1%; 20/1349
Have you actually obtained a second opinion? (multiple answers NOT possible)	
No, never	49%; 665/1349
Yes, one	26%; 349/1349
Yes, two or more	21%; 283/1349
I do not know	3%; 36/1349
No (valid) answer	1%; 16/1349
Where did you obtain the second opinion? (multiple answers possible)	
Physician in a practice	62%; 392/632
Physician in a hospital	46%; 290/632
Via the health insurer	4%; 27/632
Via an online portal	2%; 12/632
Other	7%; 45/632
No (valid) answer	1%; 4/632
To what extent did the most recent second opinion contribute to your decision? (multiple answers NOT possible)	
I was sure which treatment to choose after obtaining the second opinion	84%; 528/632
I still was not sure which treatment to choose after obtaining the second opinion	13%; 79/632
No (valid) answer	4%; 25/632
Situations to obtain a second opinion based on documents only instead of a personally delivered one (multiple answers NOT possible)	
Yes	15%; 208/1349
No	81%; 1090/1349
No (valid) answer	4%; 51/1349
Awareness of second opinion programs by the participant’s health insurer (multiple answers NOT possible)	
Yes	9%; 115/1349
No	90%; 1210/1349
No (valid) answer	2%; 24/1349
Awareness of second opinion provider (multiple answers NOT possible)	
Yes	9%; 123/1349
No	89%; 1199/1349
No (valid) answer	2%; 27/1349

### Experiences with SOs

Nearly half of the participants had obtained SOs in the past. A majority had obtained an SO for orthopedic conditions (30%; 187/362). Conservative and surgical treatment recommendations were equally distributed (31%; 193/632 vs. 29%; 185/632). The majority of SOs were obtained from a physician in a practice (62%; 392/632), followed by a physician in a hospital (46%; 290/632). Only 9% (57/632) obtained a further opinion after the SO. After obtaining the SO, 84% (528/632) were sure of which treatment to choose. Detailed information is shown in Table 3 and in the Supplementary material.

### Design of the SO procedure

**Fig. 2:**
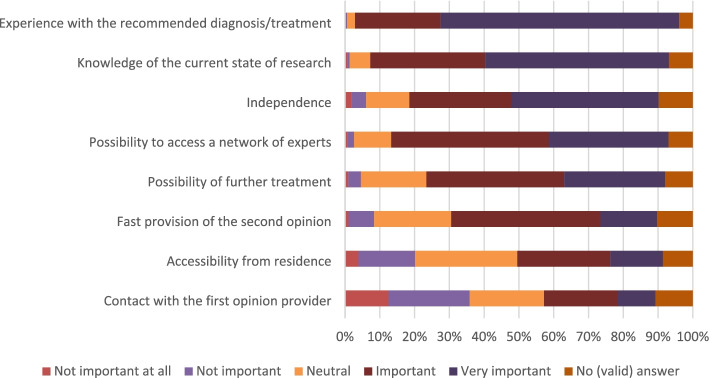
Rating of importance of qualification criteria for the physician providing an SO in case of hypothetical knee pain

The participants wanted to be informed by a physician (93%; 1279/1349) about the possibility of receiving an SO in the form of direct and personal information (89%; 1201/1349). Most participants wanted to receive information on treatment options before seeking an SO (82%; 1109/1349). Participants were asked to imagine that they have been suffering from moderate knee pain for years limiting them in their everyday life, and that they have received a recommendation for knee joint replacement. They were then asked to rate the importance of several potential qualification criteria for physicians providing SOs (see Fig. 2).

Participants were willing to travel 60 min (median; IQR 60–120) and wait 4 weeks (median; IQR 2–4). Approximately 15% indicated that there are situations in which they would more likely obtain an SO based on documents only. The most frequently mentioned situations include distance to the physician providing the SO (19%; 39/208) and time-related reasons (14%; 29/208). Detailed information can be found in Table 3 and in the Supplementary material.

### Experiences with and knowledge of SO programs by health insurers and SO providers

Few participants were aware of SO programs offered by their health insurer or by SO provider (9%; 115/1349 and 9%; 123/1349). The SO programs offered by health insurers that participants were aware of, most often related to orthopedic indications (23%; 27/115). Participants who were aware of an SO provider equally often mentioned physicians in a practice, in a hospital, or an online portal providing SOs (41%; 50/123, 39%; 48/123, and 38%; 47/123, respectively). Detailed information is shown in Table 3 and in the Supplementary material.

### Potential differences in the participants’ attitudes toward SOs based on settlement patterns

The results stratified by settlement pattern are presented in the Supplementary material. Since the stratified results were very similar, we did not perform our preplanned subgroup analysis and did not test for differences.

## Discussion

SOs are generally viewed positively and utilized by the German general population. The willingness to seek an SO is high. Most participants preferred SOs delivered within direct patient-physician contact, either in a practice or in hospital. SO programs offered by health insurers or dedicated SO providers seem to play only a minor role, as very few respondents are aware of them.

A medical record analysis and a telephone survey with a representative sample from Israel found that 14.9 and 17.2% of participants, respectively, had obtained an SO during a 1.5-year period [25]. In our sample, we found a higher proportion of people obtaining one or more SOs (47%). However, because we did not limit our question to a specific time period, the results are not comparable. A systematic review investigating oncological SOs found high heterogeneity in the rate of those who had already obtained an SO [8]. The high heterogeneity might reflect differences in focus and research methods of the included studies making a direct comparison with our results difficult. On a national level, we found a high degree of acceptance of SOs, particularly in comparison to an earlier survey of the German general population [10].

The preferred way of SO delivery was via direct and personal patient-physician contact. This is in accordance with the SO Directive, which mandates that the SO is provided verbally. Furthermore, this is comparable to another survey [10]. However, a subgroup of 15% stated that there are situations in which they would obtain an SO based on documents only instead of a personally delivered SO.

We were unable to find considerable deviations in sociodemographic characteristics between our participants and two previous surveys of patients who had obtained SOs based on documents only [20, 26]. This would have allowed us to further characterize the subgroup of patients for whom a telemedical SO might be more attractive. We were also unable to characterize patient subgroups in a related, published survey, because patient characteristics were not reported. The sample consisted of US patients who obtained an SO based on documents only, which was subsequently discussed with the patients [27]. Given the high satisfaction of the customers of the online portal [20], the SO Directive might exclude the needs of a patient subgroup by limiting SOs to those delivered verbally. However, we are currently unable to specify to which patients this would apply.

We found that participants were willing to wait about a month and travel for an hour to obtain an SO. This high willingness was documented in the context of the hypothetical knee pain described in the questionnaire. It remains unclear to what extent participants would have different views in the case of other complaints, or even in the presence of real complaints.

Our results confirm findings by Geraedts et al. on the importance of seeking an SO for different diagnoses. Participants rated obtaining an SO following a cancer diagnosis to be more important compared to diseases of bones, joints, and muscles. Although we found a wide range of indications for which participants had obtained an SO in the past, most of those were obtained for orthopedic indications (30%) and significantly fewer for oncologic indications (8%). At first glance, this contradicts the finding that oncological SOs are rated to be more important than orthopedic SOs. The high proportion of orthopedic SOs may be related to the fact that the quality of medical indications for orthopedic surgery has been discussed controversially in the media [28, 29]. This may have raised awareness and created a certain degree of public mistrust. Perhaps as a result, orthopedic SOs are common [10, 16, 17]. Furthermore, the numbers may reflect the prevalence of the indications. We found that SOs for conservative and surgical treatment recommendations were equally distributed. This shows that the patients’ need for SOs goes beyond surgical treatment recommendations and includes nonsurgical treatments. This is particularly interesting because the SO Directive only refers to elective surgical treatment recommendations and explicitly excludes oncological indications. The exclusion seems to contradict patients’ needs. Systematic review data show that internationally, SOs are often obtained for indications not currently covered by the German SO Directive. Many SOs are provided for oncological indications and elective surgery, but also for general medical concerns [8, 9]. Overall, indications rated as important by patients have not been included in the SO Directive to date, while some indications included in the SO Directive are less important from the patient perspective. The list of indications has been extended gradually in the past and will probably be expanded further in the future. The patients’ perspective should be considered when selecting further indications to include in future versions of the SO Directive .

In the context of hypothetical knee pain, participants rated experience with the recommended diagnosis/treatment, knowledge of the current state of research, and independence to be (very) important qualifying factors for the physician providing an SO by 93, 86, and 72%, respectively. These criteria are part of the SO Directive. However, 80% of participants rated the access a physician providing the SO has to a network of experts as (very) important. This is only part of the SO Directive for a subset of indications but it is part of some SO programs provided by health insurers [16]. Around 70% of participants would consider it (very) important that the second opinion provider can perform further treatment. This is contrary not only to many SO programs offered by health insurers [16] and to the SO Directive but also to the fact that independence was rated as (very) important by many participants. Perhaps the participants’ understanding of independence was different to ours. We referred to financial aspects such as (conscious or unconscious) incentives to recommend a particular treatment which could be lower when further treatment is not permitted.

According to the SO Directive, the physician providing the first opinion must inform patients about their right to obtain an SO. Given that 93% of participants want to be informed about the possibility of obtaining an SO by a physician (followed by 56% who want to be informed by the health insurer), this is mostly in accordance with the patients’ needs. Furthermore, most participants want to be informed about the possibility of obtaining an SO in a personal and direct way (89%). This can best be achieved during the consultation with the physician providing the indication. The SO Directive requires that physicians who provide the first opinion inform patients about information available online on treatment options and about a list of physicians offering SOs. This is in accordance with patients’ wishes for information on treatment options and for lists of potential SO providers. Currently, it is unknown how many physicians fulfil the obligations set out in the SO Directive.

Finally, health insurers could certainly better promote their offer of SO programs and the SO Directive. This would be indicated particularly in view of the low awareness level of SO programs in the population. The low awareness might also explain why 43% of the participants considered seeking an SO via their health insurer, but only 4% of participants who obtained an SO in the past had done so.

### Strengths and limitations

The response rate to our survey was low, despite various strategies to increase response, such as sending a reminder and offering participation in a lottery. The number of items included in our questionnaire is a possible explanation for this (47 items). We used only one validated instrument in our questionnaire. As in a previous survey [20], we found a high proportion of people for whom no overall score for health literacy could be calculated (17%). This is probably due to the provision of a ‘do not know’ category. Because we used data provided by local registration offices, we were unable to systematically compare participants with the basic population (except for the residence classification). Therefore, we cannot exclude selection bias. Furthermore, the impact of the COVID-19 pandemic on the results of our survey remains unclear. For example, the pandemic may have influenced the comparison between different ways of SO delivery and the willingness to wait or travel for an SO.

This is a comprehensive survey on SOs, and its results help to better understand patient needs, especially in the context of other parts from our extensive project ZWEIT.

### Implications for research and practice

It is important to tailor SO programs to the patients’ needs. SO programs should be evaluated with the goal of defining specific groups and circumstances for each type of SO program. Some patients’ needs identified in this project appear contradictory. Future research should examine to what extent these needs can be reconciled. Because many participants are interested in obtaining an SO but only few are aware that SO programs are offered by health insurers or SO providers, the level of awareness of these programs should be increased.

## Conclusion

SOs are generally viewed positively. Almost half of the participants have already obtained one or more SOs in the past and the respondents valued SOs for a range of indications and procedures. Only a subset of participants would obtain an SO via the health insurer or an online portal. SO programs are generally unknown. In the further development of SO programs, the patients’ perspective should be included in the decision process. This would be particularly important when deciding on the inclusion of indications as well as on the structure of the SO procedure.

## Supplementary Information

**Additional file 1.**

## Data Availability

The datasets used and/or analyzed during the current study are available from the corresponding author on reasonable request.
