# Supplementary material for: Attitude toward second opinions in Germany – a survey of the general population
Source: BMC Health Serv Res. 2022 Jan 15;22:76. doi: 10.1186/s12913-021-07422-z (PMC8760563; doi:10.1186/s12913-021-07422-z)
Supplement: Supplementary file 1 — Additional file 1. [file 12913_2021_7422_MOESM1_ESM.docx]

Attitude toward second opinions in Germany – A survey of the general population

Authors: Nadja Könsgen^1*^, Barbara Prediger^1^, Anna Schlimbach^1^, Ana-Mihaela Bora^1^, Simone Hess^1^, Michael Caspers^1^, Dawid Pieper^1^

1) Institute for Research in Operative Medicine, Witten/Herdecke University, Germany

Supplementary material

| Item | Results (%; n/N or Median (IQR)) | | | |
| --- | --- | --- | --- | --- |
|  | Overall | Stratified (cities) | Stratified (towns and suburbs) | Stratified (rural areas) |
| Health literacy | 11 (9-14)  No (valid) answer 17%; 232/1349 | 12 (9-14)  No (valid) answer 14%; 72/511 | 11 (8-14)  No (valid) answer 18%; 65/353 | 11 (8-13)  No (valid) answer 19%; 84/443 |
| Shared decision making (multiple answers NOT possible)  The physician and I should decide together  The physician should decide but highly consider my opinion  I should decide but highly consider the physicians‘ opinion  The physician should decide by his/herself  I should decide by myself  No (valid) answer | 56%; 750/1349  20%; 265/1349  19%; 257/1349  3%; 40/1349  1%; 18/1349  1%; 19/1349 | 53%; 269/511  21%; 109/511  19%; 99/511  4%; 19/511  2%; 8/511  1%; 7/511 | 54%; 189/353  21%; 73/353  20%; 72/353  3%; 9/353  1%; 5/353  1%; 5/353 | 60%; 267/443  17%; 77/443  18%; 80/443  2%; 11/443  1%; 3/443  1%; 5/443 |
| Mostly used way of travel to the participant’s general practitioner (multiple answers NOT possible)  Car  Pedestrian  Bicycle  Bus/train  Other  No (valid) answer | 46%; 625/1349  22%; 294/1349  12%; 160/1349  5%, 69/1349  2%; 26/1349  13%; 175/1349 | 32%; 166/511  29%; 147/511  13%; 67/511  8%; 43/511  3%; 14/511  14%; 74/511 | 44%; 155/353  21%; 73/353  16%; 56/353  5%; 16/353  1%; 2/353  14%; 51/353 | 64%; 285/443  14%; 63/443  8%; 37/443  2%; 8/443  2%; 8/443  9%; 42/443 |
| Travel time to the participant’s general practitioner (minutes) | 10 (5-15)  No (valid) answer  2%; 28/1349 | 10 (5-15)  No (valid) answer  2%; 10/511 | 10 (5-15)  No (valid) answer  2%; 6/353 | 10 (10-20)  No (valid) answer 2%; 8/443 |
| Is the nearest general practitioner the participant’s general practitioner? (multiple answers NOT possible)  Yes  No  No (valid) answer | 57%; 772/1349  40%; 533/1349  3%; 44/1349 | 52%; 266/511  44%; 226/511  4%; 19/511 | 55%; 193/353  43%; 152/353  2%; 8/353 | 64%; 284/443  33%; 145/443  3%; 14/443 |
| Travel time to the participant’s general practitioner, if the nearest general practitioner is not the participant’s general practitioner (minutes) | 16 (10-25)  No (valid) answer 4%; 20/533 | 18.75 (10-30)  No (valid) answer 4%; 10/226 | 15 (10-20)  No (valid) answer 3%; 4/152 | 20 (10,5-30)  No (valid) answer 4%; 6/145 |
| Travel time to the nearest hospital (multiple answers NOT possible)  Less than 15 minutes  15 to 20 minutes  20 to 30 minutes  More than 30 minutes  No (valid) answer | 37%; 504/1349  32%; 433/1349  21%; 280/1349  7%; 96/1349  3%; 36/1349 | 45%; 230/511  39%; 197/511  9%; 48/511  3%; 16/511  4%; 20/511 | 62%; 218/353  25%; 88/353  10%; 34/353  3%; 9/353  1%; 4/353 | 11%; 49/443  31%; 138/443  41%; 182/443  15%; 67/443  2%; 7/443 |
| Have you considered seeking a second opinion in the past? (multiple answers NOT possible)  Yes, twice or more  No, never  Yes, once  I do not know  No (valid) answer | 38%; 519/1349  33%; 446/1349  25%; 341/1349  3%; 35/1349  1%; 8/1349 | 40%; 203/511  32%; 165/511  26%; 131/511  2%; 10/511  0%; 2/511 | 39%; 139/353  34%; 121/353  24%; 83/353  3%; 10/353  0%; 0/353 | 36%; 159/443  33%; 146/443  27%; 121/443  3%; 13/443  1%; 4/443 |
| For which diagnoses would you find it important to obtain a second opinion? (multiple answers possible)  Cancer  Diseases of bones, joints, muscles  Disease of internal organs  Neurological diseases  Disease of the mind / psyche  Other  No (valid) answer | 78%; 1049/1349  58%; 784/1349  56%; 758/1349  46%; 621/1349  36%; 491/1349  12%; 164/1349  2%; 29/1349 | 77%; 392/511  59%; 300/511  58%; 297/511  50%; 258/511  38%; 193/511  15%; 76/511  1%; 7/511 | 80%; 282/353  59%; 208/353  52%; 184/353  42%; 147/353  33%; 116/353  10%; 37/353  1%; 5/353 | 78%; 344/443  58%; 255/443  58%; 258/443  44%; 194/443  38%; 168/443  10%; 46/443  3%; 12/443 |
| For which examinations or treatments would it be important for you to obtain a second opinion? (multiple answers possible)  Drug treatment of cancer  Chemotherapy  Bone/joint surgery  Radiotherapy  Surgery on the internal organs  Cardiac catheter  Drug treatment of another disease than cancer  Dental prosthesis  Dental surgeries Arthroscopy  Endoscopy  Other  No (valid) answer | 66%; 893/1349  65%; 872/1349  59%; 797/1349  56%; 750/1349  51%; 684/1349  27%; 358/1349  22%; 300/1349  21%; 283/1349  20%; 270/1349  17%; 233/1349  16%; 222/1349  6%; 80/1349  3%; 37/1349 | 68%; 347/511  65%; 332/511  63%; 322/511  55%; 282/511  55%; 281/511  31%; 158/511  23%; 117/511  23%; 118/511  24%; 124/511  20%; 104/511  16%; 80/511  6%; 32/511  2%; 12/511 | 63%; 224/353  64%; 225/353  57%; 202/353  56%; 197/353  46%; 161/353  22%; 77/353  23%; 81/353  20%; 72/353  17%; 61/353  15%; 52/353  17%; 61/353  6%; 20/353  3%; 10/353 | 67%; 295/443  66%; 292/443  57%; 251/443  56%; 246/443  50%; 223/443  26%; 114/443  21%; 94/443  19%; 83/443  18%; 80/443  16%; 69/443  16%; 73/443  5%; 22/443  2%; 11/443 |
| Assessment of the importance of a second opinion for specific therapeutic interventions (multiple answers NOT possible)  Disc surgery  Not important at all  Not important  Neutral  Important  Very important  No (valid) answer  Joint replacement  Not important at all  Not important  Neutral  Important  Very important  No (valid) answer  Prostatectomy  Not important at all  Not important  Neutral  Important  Very important  No (valid) answer  Hysterectomy  Not important at all  Not important  Neutral  Important  Very important  No (valid) answer  Meniscectomy/ meniscus resection  Not important at all  Not important  Neutral  Important  Very important  No (valid) answer  Shoulder arthroscopy  Not important at all  Not important  Neutral  Important  Very important  No (valid) answer  Tonsillectomy/tonsillotomy  Not important at all  Not important  Neutral  Important  Very important  No (valid) answer  Mole removal  Not important at all  Not important  Neutral  Important  Very important  No (valid) answer  Appendectomy  Not important at all  Not important  Neutral  Important  Very important  No (valid) answer  Wisdom tooth removal  Not important at all  Not important  Neutral  Important  Very important  No (valid) answer | 2%; 21/1349  5%; 73/1349  17%; 223/1349  32%; 427/1349  40%; 540/1349  5%: 65/1349  2%; 24/1349  5%; 61/1349  16%; 212/1349  38%; 510/1349  35%; 477/1349  5%; 65/1349  4%; 51/1349  6%; 81/1349  18%; 249/1349  30%; 410/1349  26%; 357/1349  15%; 201/1349  5%; 65/1349  9%; 115/1349  21%; 290/1349  23%; 316/1349  21%; 287/1349  20%; 276/1349  4%; 56/1349  13%; 177/1349  31%; 412/1349  26%; 354/1349  17%; 223/1349  9%; 127/1349  6%; 82/1349  21%; 281/1349  34%; 465/1349  18%; 249/1349  9%; 115/1349  12%; 157/1349  17%; 226/1349  35%; 473/1349  23%; 312/1349  9%; 128/1349  4%; 55/1349  11%; 155/1349  23%; 315/1349  35%; 473/1349  18%; 249/1349  9%; 115/1349  3%; 44/1349  11%; 153/1349  27%; 368/1349  39%; 527/1349  14%; 187/1349  6%; 79/1349  3%; 43/1349  11%; 145/1349  27%; 363/1349  38%; 515/1349  16%; 214/1349  6%; 77/1349  3%; 36/1349  11%; 144/1349 | 2%; 8/511  6%; 29/511  15%; 78/511  32%; 165/511  41%; 210/511  4%; 21/511  2%; 11/511  4%; 21/511  14%; 74/511  39%; 201/511  37%; 189/511  3%; 15/511  3%; 16/511  6%; 29/511  20%; 101/511  31%; 156/511  26%; 135/511  14%; 74/511  5%; 27/511  7%; 38/511  21%; 105/511  24%; 124/511  23%; 115/511  20%; 102/511  4%; 18/511  12%; 61/511  32%; 164/511  26%; 134/511  19%; 95/511  8%; 39/511  7%; 36/511  19%; 99/511  33%; 170/511  19%; 98/511  10%; 53/511  11%; 55/511  18%; 90/511  33%; 169/511  23%; 118/511  11%; 55/511  5%; 24/511  11%; 55/511  25%; 127/511  35%; 177/511  17%; 89/511  10%; 51/511  3%; 16/511  10%; 51/511  29%; 150/511  36%; 185/511  15%; 76/511  7%; 35/511  3%; 14/511  10%; 51/511  29%; 149/511  37%; 190/511  15%; 78/511  5%; 27/511  2%; 12/511  11%; 55/511 | 1%; 2/353  5%; 17/353  18%; 64/353  30%; 107/353  41%; 144/353  5%; 19/353  2%; 6/353  5%; 16/353  17%; 60/353  35%; 123/353  36%; 127/353  6%; 21/353  4%; 13/353  8%; 27/353  17%; 60/353  31%; 108/353  26%; 91/353  15%; 54/353  6%; 20/353  9%; 32/353  22%; 78/353  22%; 79/353  19%; 68/353  22%; 76/353  4%; 14/353  11%; 40/353  31%; 110/353  27%; 96/353  15%; 53/353  11%; 40/353  5%; 19/353  24%; 83/353  35%; 122/353  16%; 57/353  8%; 29/353  12%; 43/353  18%; 63/353  39%; 136/353  20%; 70/353  7%; 26/353  4%; 15/353  12%; 43/353  22%; 79/353  35%; 123/353  21%; 74/353  7%; 25/353  3%; 10/353  12%; 42/353  26%; 92/353  40%; 141/353  15%; 54/353  4%; 15/353  3%; 10/353  12%; 41/353  25%; 89/353  38%; 135/353  16%; 56/353  6%; 20/353  3%; 11/353  12%; 42/353 | 2%; 8/443  6%; 27/443  18%; 78/443  32%; 143/443  38%; 168/443  4%; 19/443  2%; 7/443  5%; 24/443  16%; 72/443  39%; 173/443  33%; 144/443  5%; 23/443  5%; 21/443  5%; 22/443  18%; 80/443  31%; 137/443  27%; 120/443  14%; 63/443  4%; 17/443  9%; 41/443  22%; 98/443  24%; 105/443  21%; 95/443  20%; 87/443  5%; 23/443  17%; 74/443  29%; 127/443  26%; 116/443  14%; 63/443  9%; 40/443  6%; 26/443  22%; 96/443  36%; 160/443  19%; 83/443  6%; 28/443  11%; 50/443  16%; 70/443  35%; 154/443  26%; 115/443  9%; 41/443  3%; 15/443  11%; 48/443  23%; 103/443  36%; 160/443  17%; 77/443  8%; 34/443  4%; 18/443  12%; 51/443  26%; 116/443  42%; 186/443  12%; 52/443  6%; 26/443  4%; 18/443  10%; 45/443  26%; 115/443  40%; 176/443  16%; 72/443  6%; 28/443  3%; 13/443  9%; 39/443 |
| Where can you generally imagine seeking a second opinion? (multiple answers possible)  Physician in a practice  Physician in a hospital  Via the health insurer  Via an online portal  Other  No (valid) answer | 81%; 1090/1349  72%; 973/1349  43%; 577/1349  16%; 221/1349  4%; 52/1349  1%; 20/1349 | 84%; 429/511  72%; 370/511  44%; 223/511  14%; 73/511  4%; 22/511  1%; 5/511 | 81%; 287/353  75%; 263/353  40%; 142/353  18%; 62/353  2%; 8/353  1%; 4/353 | 77%; 343/443  71%; 314/443  43%; 191/443  19%; 84/443  4%; 19/443  1%; 6/443 |
| Based on your regional health care situation, where would you seek a second opinion? (multiple answers possible)  Physician in a practice  Physician in a hospital  Via the health insurer  Via an online portal  Other  No (valid) answer | 78%; 1050/1349  66%; 887/1349  37%; 502/1349  13%; 181/1349  4%; 48/1349  2%; 23/1349 | 80%; 411/511  66%; 338/511  38%; 196/511  11%; 58/511  4%; 20/511  1%; 6/511 | 79%; 278/353  68%; 241/353  35%; 125/353  13%; 47/353  3%; 9/353  1%; 3/353 | 74%; 329/443  63%; 281/443  36%; 161/443  16%; 73/443  4%; 18/443  2%; 10/443 |
| Assessment of the different options for obtaining a second opinion (multiple answers NOT possible)  Personally delivered second opinion  Considering  Tending to consider  Neutral  Tending not to consider  Not considering  No (valid) answer  Second opinion delivered via phone  Considering  Tending to consider  Neutral  Tending not to consider  Not considering  No (valid) answer  Second opinion based on documents only  Considering  Tending to consider  Neutral  Tending not to consider  Not considering  No (valid) answer | 88%; 1184/1349  9%; 121/1349  2%; 23/1349  0%; 3/1349  0%; 2/1349  1%; 16/1349  13%; 182/1349  10%; 132/1349  24%; 318/1349  21%; 283/1349  18%; 249/1349  14%; 185/1349  10%; 132/1349  6%; 87/1349  17%; 223/1349  23%; 309/1349  29%; 395/1349  15%; 203/1349 | 90%; 461/511  8%; 39/511  1%; 7/511  0%; 1/511  0%; 0/511  1%; 3/511  13%; 65/511  12%; 63/511  23%; 120/511  22%; 114/511  17%; 88/511  12%; 61/511  10%; 49/511  6%; 31/511  16%; 80/511  22%; 112/511  33%; 170/511  14%; 69/511 | 87%; 306/353  10%; 37/353  2%; 6/353  0%; 0/353  0%; 1/353  1%; 3/353  14%; 50/353  8%; 30/353  24%; 83/353  20%; 71/353  20%; 69/353  14%; 50/353  11%; 40/353  7%; 23/353  15%; 52/353  25%; 90/353  27%; 94/353  15%; 54/353 | 87%; 384/443  9%; 42/443  2%; 9/443  0%; 2/443  0%; 1/443  1%; 5/443  15%; 65/443  9%; 38/443  24%; 105/443  20%; 90/443  19%; 84/443  14%; 61/443  9%; 41/443  7%; 33/443  19%; 85/443  23%; 101/443  27%; 119/443  14%; 64/443 |
| Comparison of ratings for SOs within direct and personal patient-physician contact and SOs based on documents only (multiple answers NOT possible)  SOs within direct and personal patient-physician contact rated better  Both rated equally  SOs based on documents only rated better  No (valid) answer | 73%; 991/1349  10%; 133/1349  1%; 19/1349  15%; 206/1349 | 76%: 349/511  9%; 47/511  1%; 6/511  14%; 69/511 | 71%; 251/353  12%; 42/353  1%; 4/353  16%; 56/353 | 74%; 327/443  10%; 43/443  2%; 9/443  14%; 64/443 |
| Have you actually obtained a second opinion? (multiple answers NOT possible)  No, never  Yes, one  Yes, two or more  I do not know  No (valid) answer | 49%; 665/1349  26%; 349/1349  21%; 283/1349  3%; 36/1349  1%; 16/1349 | 48%; 246/511  27%; 137/511  22%; 112/511  2%; 12/511  1%; 4/511 | 47%; 165/353  26%; 92/353  23%; 80/353  4%; 13/353  1%; 3/353 | 53%; 237/443  26%; 113/443  18%; 78/443  2%; 10/443  1%; 5/443 |
| Condition for which a second opinion was obtained (in case of multiple opinions reference to the most recent one) (multiple answers NOT possible)  Orthopedics  Dentistry  Internal medicine  Oncology  Gynecology  Neurology/neurosurgery  Cardiology  Dermatology  Ear, nose and throat  Ophthalmology  Urology  General and visceral medicine  Pediatrics  Another indication  No (valid) answer | 30%; 187/362  10%; 62/632  9%; 56/632  8%; 48/632  5%; 31/632  4%; 24/632  4%; 23/632  3%; 20/632  3%; 17/632  2%; 15/632  2%; 13/632  1%; 9/632  1%; 5/632  5%; 32/632  14%; 90/632 | 29%; 72/249  12%; 30/249  12%; 29/249  10%; 24/249  4%; 10/249  4%; 9/10  4%; 9/10  2%; 5/249  4%; 10/249  2%; 6/249  1%; 3/249  1%; 2/249  0%; 1/249  4%; 10/249  12%; 29/249 | 27%; 47/172  10%; 18/172  8%; 14/172  5%; 8/172  5%; 9/172  6%; 10/172  4%; 7/172  4%; 7/172  2%; 4/172  2%; 3/172  3%; 5/172  1%; 2/172  2%; 3/172  6%; 10/172  15%; 25/172 | 34%; 64/191  7%; 14/191  6%; 12/191  8%; 15/191  6%; 12/191  3%; 5/191  3%; 6/191  4%; 7/191  2%; 3/191  3%; 5/191  3%; 5/191  3%; 5/191  1%; 1/191  6%; 11/191  14%; 26/191 |
| Treatment recommendation for which a second opinion was obtained (in case of multiple opinions reference to the most recent one) (multiple answers NOT possible)  Conservative  Surgical  Mixed  Unclear  No (valid) answer | 31%; 193/632  29%; 185/632  7%; 44/632  9%; 54/632  25%; 156/632 | 34%; 84/249  27%; 67/249  6%; 15/249  10%; 24/249  24%; 59/249 | 30%; 52/172  31%; 54/172  8%; 14/172  9%; 15/172  22%; 37/172 | 30%; 57/191  31%; 59/191  8%; 15/191  8%; 15/191  24%; 45/191 |
| Reason for obtaining the second opinion (in case of multiple opinions reference to the most recent one) (multiple answers possible)  Need for more information on further treatment recommendations  Confirmation that the diagnosis was correct  Need for more information on the diagnosis  Confirmation that the initial treatment recommendation was correct  Wish for another treatment recommendation  Dissatisfaction with the consultation with the first opinion provider  Need for more information on the initial treatment recommendation  Lack of trust in the first opinion provider  Recommendation by relatives/friends or the first opinion provider  Wish for another diagnosis  Another reason  No (valid) answer | 49%; 307/632  44%; 277/632  40%; 252/632  36%; 229/632  29%; 185/632  27%; 168/632  26%; 166/632  24%; 149/632  13%; 80/632  9%; 60/632  9%; 58/632  1%; 4/632 | 49%; 121/249  38%; 94/249  40%; 100/249  38%; 95/249  33%; 83/249  27%; 67/249  27%; 66/249  26%; 65/249  13%; 33/249  11%; 28/249  10%; 26/249  0%; 0/249 | 52%; 89/172  49%; 85/172  41%; 70/172  34%; 59/172  28%; 49/172  27%; 46/172  25%; 43/172  21%; 36/172  12%; 21/172  9%; 16/172  9%; 15/172  1%; 1/172 | 45%; 86/191  47%; 90/191  38%; 72/191  36%; 69/191  25%; 47/191  28%; 53/191  26%; 49/191  23%; 43/191  14%; 26/191  8%; 16/191  8%; 15/191  1%; 2/191 |
| Where did you obtain the (most recent) second opinion? (multiple answers possible)  Physician in a practice  Physician in a hospital  Via the health insurer  Via an online portal  Other  No (valid) answer | 62%; 392/632  46%; 290/632  4%; 27/632  2%; 12/632  7%; 45/632  1%; 4/632 | 65%; 161/249  42%; 104/249  6%; 15/249  2%; 5/249  9%; 23/249  0%; 1/249 | 64%; 110/172  41%; 70/172  3%; 6/172  3%; 6/172  5%; 9/172  1%; 2/172 | 55%; 106/191  55%; 106/191  2%; 4/191  1%; 1/191  6%; 12/191  0%; 0/191 |
| Did the most recent second opinion confirm the treatment recommendation of the first opinion? (multiple answers NOT possible)  Yes  Partially  No  No (valid) answer | 40%; 252/632  30%; 189/632  28%; 179/632  2%; 12/632 | 37%; 92/249  31%; 77/249  30%; 75/249  2%; 5/249 | 39%; 67/172  31%; 54/172  27%; 47/172  2%; 4/172 | 45%; 86/191  27%; 51/191  27%; 52/191  1%; 2/191 |
| To what extent did the most recent second opinion contribute to your decision? (multiple answers NOT possible)  I was sure which treatment to choose after obtaining the second opinion  I still was not sure which treatment to choose after obtaining the second opinion  No (valid) answer | 84%; 528/632  13%; 79/632  4%; 25/632 | 83%; 206/249  14%; 36/249  3%; 7/249 | 82%; 141/172  13%; 23/172  5%; 8/172 | 86%; 165/191  9%; 18/191  4%; 8/191 |
| Did you obtain another opinion after the second opinion? (multiple answers NOT possible)  Yes  No  No (valid) answer | 9%; 57/632  89%; 564/632  2%; 11/632 | 8%; 20/249  90%; 225/249  2%; 4/249 | 9%; 16/172  88%; 152/172  2%; 4/172 | 9%; 18/191  90%; 171/191  1%; 2/191 |
| From whom would you like to be informed about the provision of a second opinion? (multiple answers possible)  Physicians  Health insurer  Consumer center  Other  No (valid) answer | 93%; 1249/1349  56%; 749/1349  13%; 182/1349  4%; 48/1349  2%; 23/1349 | 93%; 473/511  54%; 277/511  16%; 80/511  4%; 22/511  1%; 5/511 | 94%; 333/353  52%; 183/353  12%; 41/353  3%; 10/353  1%; 5/353 | 93%; 411/443  61%; 270/443  12%; 53/443  3%; 15/443  1%; 5/443 |
| How would you like to receive information about the provision of a second opinion? (multiple answers possible)  Direct/personal information  Information leaflet  Via a webpage  Insurance magazine  Social media  Other  No (valid) answer | 89%; 1201/1349  38%; 518/1349  27%; 370/1349  11%; 151/1349  5%; 68/1359  2%; 28/1349  2%; 29/1349 | 91%; 464/511  40%; 203/511  29%; 146/511  12%; 62/511  5%; 27/511  2%; 9/511  1%; 7/511 | 88%; 310/353  37%; 129/353  27%; 95/353  10%; 34/353  4%; 15/353  2%; 7/353  2%; 6/353 | 89%; 395/443  39%; 174/443  26%; 117/443  12%; 53/443  5%; 22/443  2%; 11/443  2%; 7/443 |
| What information would you like to have in advance before seeking a second opinion? (multiple answers possible)  Information on treatment options  List of potential second opinion providers  Overview on potentially arising costs  Detailed information on second opinions  Other  No (valid) answer | 82%; 1109/1349  68%; 919/1349  39%; 526/1349  30%; 399/1349  2%; 24/1349  2%; 31/1349 | 82%; 421/511  67%; 342/511  42%; 213/511  32%; 164/511  2%; 11/511  1%; 6/511 | 83%; 292/353  66%; 232/353  38%; 135/353  29%; 102/353  1%; 5/353  2%; 6/353 | 83%; 369/443  73%; 324/443  38%; 168/443  27%; 120/443  2%; 8/443  2%; 8/443 |
| Hypothetical knee pain: assessment of various aspects in the choice for a second opinion provider (multiple answers NOT possible)  Experience with the recommended diagnosis/treatment  Not important at all  Not important  Neutral  Important  Very important  No (valid) answer  Knowledge of the current state of research  Not important at all  Not important  Neutral  Important  Very important  No (valid) answer  Independence  Not important at all  Not important  Neutral  Important  Very important  No (valid) answer  Possibility to access a network of experts  Not important at all  Not important  Neutral  Important  Very important  No (valid) answer  Possibility of further treatment  Not important at all  Not important  Neutral  Important  Very important  No (valid) answer  Fast provision of the second opinion  Not important at all  Not important  Neutral  Important  Very important  No (valid) answer  Accessibility from residence  Not important at all  Not important  Neutral  Important  Very important  No (valid) answer  Contact with the first opinion provider  Not important at all  Not important  Neutral  Important  Very important  No (valid) answer | 0%; 1/1349  1%; 7/1349  2%; 31/1349  25%; 332/1349  69%; 925/1349  4%; 53/1349  1%; 9/1349  1%; 9/1349  6%; 80/1349  33%; 446/1349  53%; 712/1349  7%; 93/1349  2%; 26/1349  4%; 56/1349  12%; 168/1349  29%; 396/1349  42%; 571/1349  10%; 132/1349  1%; 11/1349  2%; 24/1349  11%; 144/1349  45%; 612/1349  34%; 464/1349  7%; 94/1349  1%; 13/1349  4%; 49/1349  19%; 254/1349  40%; 534/1349  29%; 391/1349  8%; 108/1349  1%; 14/1349  7%; 99/1349  22%; 299/1349  43%; 579/1349  16%; 219/1349  10%; 139/1349  4%; 52/1349  16%; 220/1349  29%; 396/1349  27%; 361/1349  15%; 204/1349  9%; 116/1349  13%; 169/1349  23%; 315/1349  21%; 288/1349  21%; 287/1349  11%; 145/1349  11%; 145/1349 | 0%; 1/511  1%; 4/511  3%; 13/511  24%; 123/511  69%; 355/511  3%; 15/511  1%; 4/511  1%; 3/511  6%; 32/511  32%; 161/511  55%; 283/511  5%; 28/511  3%; 15/511  3%; 15/511  11%; 58/511  29%; 148/511  45%; 228/511  9%; 47/511  0%; 1/511  2%; 8/511  11%; 58/511  46%; 237/511  34%; 175/511  6%; 32/511  1%; 4/511  4%; 19/511  22%; 112/511  37%; 188/511  29%; 149/511  8%; 39/511  2%; 8/511  9%; 48/511  24%; 124/511  40%; 202/511  15%; 76/511  10%; 53/511  4%; 22/511  17%; 87/511  29%, 150/511  28%; 145/511  13%; 68/511  8%; 39/511  15%; 75/511  24%; 121/11  22%; 111/511  20%; 104/511  10%; 51/511  10%; 49/511 | 0%; 0/353  1%; 3/353  3%; 9/353  22%; 79/353  70%; 248/353  4%; 14/353  1%; 4/353  1%; 4/353  6%; 21/353  35%; 122/353  52%; 182/353  6%; 20/353  1%; 5/353  5%; 19/353  15%; 54/353  30%; 106/353  39%; 138/353  9%; 31/353  1%; 4/353  2%; 7/353  12%; 43/353  47%; 165/353  32%; 114/353  6%; 20/353  1%; 5/353  3%; 10/353  16%; 57/353  44%; 157/353  28%; 100/353  7%; 24/353  1%; 4/353  6%; 20/353  23%; 80/353  46%; 162/353  16%; 56/353  9%; 31/353  4%; 14/353  14%; 51/353  31%; 111/353  27%; 96/353  14%; 50/353  9%; 31/353  11%; 40/353  24%; 86/353  23%; 82/353  21%; 74/353  9%; 33/353  11%; 38/353 | 0%; 0/443  0%; 0/443  2%; 8/443  26%; 116/443  68%; 303/443  4%; 16/443  0%; 1/443  0%; 2/443  6%; 25/443  34%; 152/443  52%; 230/443  7%; 33/443  1%; 4/443  5%; 20/443  12%; 54/443  30%; 131/443  44%; 194/443  9%; 40/443  1%; 6/443  2%; 8/443  9%; 41/443  44%; 194/443  37%; 163/443  7%; 31/443  1%; 4/443  4%; 18/443  18%; 78/443  40%; 176/443  30%; 134/443  7%; 33/443  0%; 2/443  7%; 30/443  19%; 86/443  46%; 202/443  19%; 82/443  9%; 41/443  3%; 15/443  18%; 79/443  27%; 121/443  26%; 115/443  18%; 81/443  7%; 32/443  12%; 52/443  21%; 95/443  21%; 92/443  23%; 103/443  13%; 57/443  10%; 44/443 |
| Hypothetical knee pain: willingness to travel (minutes) | 60 (60-120)  No (valid) answer 7%; 95/1349 | 60 (60-120)  No (valid) answer 8%; 39/511 | 60 (60-120)  No (valid) answer 5%; 17/353 | 60 (60-120)  No (valid) answer 6%; 28/443 |
| Hypothetical knee pain: willingness to wait (weeks) | 4 (2-4)  No (valid) answer 5%; 64/1349 | 4 (2-4)  No (valid) answer 6%; 30/511 | 3,25 (2-4)  No (valid) answer 3%; 9/353 | 3,5 (2-4)  No (valid) answer 3%; 13/443 |
| Situations to obtain a second opinion based on documents only instead of a personally delivered one (multiple answers NOT possible)  Yes  No  No (valid) answer | 15%; 208/1349  81%; 1090/1349  4%; 51/1349 | 16%; 80/511  81%; 416/511  3%; 15/511 | 18%; 64/353  78%; 277/353  3%; 12/353 | 12%; 54/443  84%; 374/443  3%, 15/443 |
| If yes, type of situation (multiple answers possible)  Distance to the second opinion physician  Time-related reasons  Complex issues (urgency)  Practical reasons  Available documents sufficient  Specialized / reputable physician  Legal reasons (lawsuit)  Simple issues  Financial reasons  Other reasons  No (valid) answer | 19%; 39/208  14%; 29/208  7%; 15/208  7%; 15/208  6%; 13/208  6%; 12/208  5%; 11/208  3%; 7/208  1%; 3/208  4%; 8/208  42%; 87/208 | 23%; 18/80  20%; 16/80  9%; 7/80  10%; 8/80  8%; 6/80  5%; 4/80  4%; 3/80  4%; 3/80  3%; 2/80  4%; 3/80  33%; 26/80 | 20%; 13/64  13%; 8/64  6%; 4/64  5%; 3/64  5%; 3/64  6%; 4/64  9%; 6/64  3%; 2/64  2%: 1/64  2%; 1/64  45%; 29/64 | 15%; 8/54  9%; 5/54  4%; 2/54  7%; 4/54  6%; 3/54  7%; 4/54  4%; 2/54  4%; 2/54  0%; 0/54  7%; 4/54  46%; 25/54 |
| Awareness of second opinion programs by the participant’s health insurer (multiple answers NOT possible)  Yes  No  No (valid) answer | 9%; 115/1349  90%; 1210/1349  2%; 24/1349 | 8%; 43/511  91%; 463/511  1%; 5/511 | 10%; 36/353  89%; 313/353  1%; 4/353 | 7%; 30/443  92%; 409/443  1%; 4/443 |
| If yes, indications for which second opinion programs are provided (multiple answers possible)  Orthopedics  Dentistry  No restriction  Oncology  Gynecology  Ear, nose and throat  Cardiology  Ophthalmology  Dermatology  Gastroenterology  Pediatrics  Urology  Another indication  I do not know  No (valid) answer | 23%; 27/115  10%; 12/115  6%; 7/115  6%; 7/115  5%; 6/115  4%; 5/115  3%; 4/115  3%; 4/115  3%; 3/115  1%; 1/115  1%; 1/115  1%; 1/115  14%; 16/115  8%; 9/115  32%; 37/115 | 33%; 14/43  9%; 4/43  7%; 3/43  7%; 3/43  7%; 3/43  7%; 3/43  2%; 1/43  7%; 3/43  7%; 3/43  2%; 1/43  0%; 0/43  0%; 0/43  12%; 5/43  7%; 3/43  26%; 11/43 | 19%; 7/36  11%; 4/36  8%; 3/36  0%; 0/36  6%; 2/36  6%; 2/36  0%; 0/36  0%; 0/36  0%; 0/36  0%; 3/36  0%; 0/36  3%; 1/36  17%; 6/36  8%; 3/36  33%; 12/36 | 20%; 6/30  13%; 4/30  3%; 1/30  10%; 3/30  3%; 1/30  0%; 0/30  7%; 2/30  3%; 1/30  0%; 0/30  0%; 0/30  3%; 1/30  0%; 0/30  17%; 5/30  10%; 3/30  33%; 10/30 |
| If yes, second opinion programs free of costs? (multiple answers NOT possible)  Yes  Partially  No  I do not know  No (valid) answer | 68%; 78/115  7%; 8/115  2%; 2/115  19%; 22/115  4%; 5/115 | 77%; 33/43  7%; 3/43  2%; 1/43  12%; 5/43  2%; 1/43 | 67%; 24/36  11%; 4/36  0%; 0/36  19%; 7/36  3%; 1/36 | 60%; 18/30  3%; 1/30  3%; 1/30  23%; 7/30  10%; 3/30 |
| Awareness of second opinion provider (multiple answers NOT possible)  Yes  No  No (valid) answer | 9%; 123/1349  89%; 1199/1349  2%; 27/1349 | 9%; 45/511  90%; 460/511  1%; 6/511 | 11%; 40/353  87%; 307/353  2%; 6/353 | 8%; 36/443  90%; 400/443  2%; 7/443 |
| If yes, type of second opinion provider (multiple answers possible)  Physician in a practice providing second opinions  Hospital providing second opinions  Online portal providing second opinions  Other  No (valid) answer | 41%; 50/123  39%; 48/123  38%; 47/123  15%; 19/123  2%; 2/123 | 44%; 20/45  29%; 13/45  33%; 15/45  22%; 10/45  2%; 1/45 | 30%; 12/40  45%; 18/40  45%; 18/40  5%; 2/40  0%; 0/40 | 44%; 16/36  47%; 17/36  39%; 14/36  19%; 7/36  3%; 1/36 |
| If yes, how did you get to know about the second opinion provider? (multiple answers possible)  Physician  Relatives/friends  Internet  Health insurer  Other  No (valid) answer | 44%; 54/123  39%; 48/123  37%; 45/123  24%; 30/123  14%; 17/123  2%; 2/123 | 38%; 17/45  44%; 20/45  38%; 17/45  31%; 14/45  16%; 7/45  2%; 1/45 | 48%; 19/40  45%; 18/40  43%; 17/40  15%; 6/40  10%; 4/40  0%; 0/40 | 50%; 18/36  25%; 9/36  28%; 10/36  25%; 9/36  17%; 6/36  3%; 1/36 |
| Gender (multiple answers NOT possible)  Female  Male  Diverse  No (valid) answer | 56%; 758/1349  43%; 580/1349  0%; 0/1349  1%; 11/1349 | 57%; 291/511  43%; 220/511  0%; 0/511  0%; 0/511 | 56%; 196/353  44%; 154/353  0%; 0/353  1%; 3/353 | 57%; 251/443  43%; 191/443  0%; 0/443  0%; 1/443 |
| Age (years) | 58 (44-69)  No (valid) answer 3%; 40/1349 | 58 (41-69)  No (valid) answer 3%; 11/511 | 58 (43,75-67)  No (valid) answer 1%; 7/353 | 58 (45-68,5)  No (valid) answer 2%; 8/443 |
| Marital status (multiple answers NOT possible)  Married/ registered partnership, living together  Single  Divorced/ registered partnership annulled  Widowed/death of registered partner  Married/ registered partnership, living apart  No (valid) answer | 58%; 787/1349  21%; 287/1349  8%; 109/1349  8%: 104/1349  2%; 24/1349  3%; 38/1349 | 55%; 283/511  25%; 127/511  8%; 40/511  8%; 41/511  2%; 12/511  2%; 8/511 | 64%; 225/353  17%; 60/353  8%; 30/353  6%; 20/353  2%; 8/353  3%; 10/353 | 59%; 262/443  21%; 94/443  8%; 35/443  8%; 37/443  1%; 4/443  2%; 11/443 |
| Sharing a household with a spouse/registered or non-martial/non-registered partner (multiple answers NOT possible)  Yes  No  No (valid) answer | 66%; 885/1349  30%; 404/1349  4%; 60/1349 | 64%; 326/511  33%; 171/511  3%; 14/511 | 67%; 237/353  28%; 98/353  5%; 18/353 | 68%; 303/443  27%; 120/443  5%; 20/443 |
| Highest general education school qualification (multiple answers NOT possible)  Student attending a fulltime general education school  Left school without any school-leaving certificate  Hauptschule leaving certificate (Volksschule leaving certificate) or equivalent degree  Polytechnische Oberschule of the German Democratic Republic with a leaving certificate from Grade 8 or Grade 9  Realschule leaving certificate (Mittlere Reife) or equivalent degree  Polytechnische Oberschule of the German Democratic Republic with a leaving certificate from Grade 10  Fachhochschulreife, leaving certificate from a Fachoberschule  Abitur/General or subject-specific higher education entrance qualification  Other school leaving certificate  No (valid) answer | 2%; 26/1349  1%; 9/1349  4%; 57/1349  4%; 48/1349  8%; 107/1349  28%; 378/1349  9%; 127/1349  36%; 489/1349  2%; 27/1349  6%; 81/1349 | 1%; 5/511  0%; 1/511  4%; 22/511  3%; 13/511  8%; 41/511  21%; 108/511  9%; 47/511  45%; 229/511  2%; 8/511  7%; 37/511 | 3%; 10/353  1%; 3/353  4%; 13/353  4%; 13/353  7%; 26/353  33%; 116/353  9%; 32/353  33%; 115/353  2%; 8/353  5%; 17/353 | 2%; 10/443  1%; 4/443  5%; 20/443  5%; 21/443  8%; 37/443  33%; 144/443  10%; 44/443  30%; 132/443  2%; 11/443  5%; 20/443 |
| Educational level (multiple answers NOT possible)  Vocational qualification ongoing  Without vocational qualification  Vocational qualification (duration at least 1 year) completed  Higher education degree  No (valid) answer | 2%; 23/1349  2%; 32/1349  55%; 740/1349  35%; 473/1349  6%; 81/1349 | 2%; 11/511  2%; 10/511  48%; 246/511  42%; 215/511  6%; 29/511 | 2%; 6/353  3%; 10/353  56%; 196/353  34%; 120/353  6%; 21/353 | 1%; 6/443  2%; 10/443  64%; 283/443  28%; 123/443  5%; 21/443 |
| Settlement pattern (multiple answers NOT possible)  Cities  Towns and suburbs  Rural areas  No (valid) answer | 38%; 511/1349  26%; 353/1349  33%; 443/1349  3%; 42/1349 | 100%; 511/511  0%; 0/511  0%; 0/511  0%; 0/511 | 0%; 0/353  100%; 353/353  0%; 0/353  0%; 0/353 | 0%; 0/443  0%; 0/443  100%; 443/443  0%; 0/443 |
| Type of health insurer (multiple answers NOT possible)  Statutory  Private  No (valid) answer | 76%; 1023/1349  7%; 98/1349  17%; 228/1349 | 79%; 405/511  8%; 42/511  13%; 64/511 | 74%; 260/353  7%; 26/353  19%; 67/353 | 76%; 338/443  7%; 29/443  17%; 76/443 |
| Number of household members (multiple answers NOT possible)  Only one person  Two or more persons  No (valid) answer | 24%; 329/1349  73%; 983/1349  3%; 37/1349 | 28%; 142/511  70%; 359/511  2%; 10/511 | 22%: 77/353  75%; 266/353  3%; 10/353 | 22%; 96/443  77%; 342/443  1%; 5/443 |
| If two or more persons, number of household members | 2 (2-3)  No (valid) answer 2%; 24/983 | 2 (2-3)  No (valid) answer 3%;11/359 | 2 (2-3)  No (valid) answer 2%; 6/266 | 2 (2-3)  No (valid) answer 2%; 7/342 |
| Number of household members older than 14 years | 2 (2-2)  No (valid) answer 28%; 382/1349 | 2 (2-2)  No (valid) answer 30%; 151/511 | 2 (2-2)  No (valid) answer 26%; 91/353 | 2 (2-2)  No (valid) answer 26%; 114/443 |
| Average net monthly income of the household (multiple answers NOT possible)  0 – 450 euro  451 – 850 euro  851 – 1000 euro  1001 – 1250 euro  1251 – 1500 euro  1501 – 1750 euro  1751 – 2000 euro  2001 – 2250 euro  2251 – 2500 euro  2501 – 2750 euro  2751 – 3000 euro  3001 – 3250 euro  3251 – 3500 euro  3501 – 3750 euro  3751 – 4000 euro  4001 – 4500 euro  4501 – 5000 euro  5001 – 5500 euro  5501 – 6000 euro  6001 – 7500 euro  7501 – 10.000 euro  10.001 – 20.000 euro  Above 20.000 euro  No (valid) answer | 2%; 21/1349  3%; 34/1349  3%; 40/1349  4%; 60/1349  6%; 83/1349  4%; 59/1349  6%; 80/1349  6%; 78/1349  6%; 86/1349  5%; 63/1349  6%; 76/1349  5%; 73/1349  4%; 58/1349  4%; 50/1349  5%; 63/1349  6%; 79/1349  4%; 57/1349  4%; 50/1349  3%; 38/1349  2%; 32/1349  2%; 22/1349  1%; 10/1349  0%; 4/1349  10%; 133/1349 | 2%; 8/511  2%; 12/511  3%; 14/511  4%; 18/511  6%; 33/511  3%; 14/511  7%; 38/511  6%; 32/511  7%; 37/511  4%; 21/511  6%; 30/511  5%; 25/511  5%; 24/511  4%; 21/511  3%; 17/511  5%; 27/511  5%; 26/511  5%; 23/511  3%; 15/511  3%; 14/511  3%; 13/511  1%; 4/511  0%; 2/511  8%; 43/511 | 1%; 4/353  2%; 7/353  3%; 9/353  3%; 11/353  5%; 19/353  5%; 16/353  5%; 19/353  7%; 23/353  7%; 24/353  6%; 21/353  5%; 17/353  4%; 13/353  4%; 14/353  3%; 12/353  6%; 21/353  7%; 25/353  5%; 16/353  5%; 16/353  4%; 13/353  3%; 9/353  1%; 5/353  2%; 6/353  0%; 0/353  9%; 33/353 | 2%; 8/443  3%; 15/443  4%; 16/443  6%; 26/443  7%; 29/443  6%; 26/443  5%; 23/443  5%; 22/443  5%; 24/443  4%; 19/443  6%; 28/443  7%; 33/443  5%; 20/443  4%; 17/443  6%; 25/443  6%; 25/443  3%; 15/443  2%; 10/443  2%; 10/443  2%; 8/443  1%; 4/443  0%; 0/443  0%; 2/443  9%; 38/443 |
